# Population Pharmacodynamics of Amphotericin B Deoxycholate for Disseminated Infection Caused by *Talaromyces marneffei*

**DOI:** 10.1128/AAC.01739-18

**Published:** 2019-01-29

**Authors:** Thuy Le, Vo Trieu Ly, Nguyen Thi Mai Thu, Ashley Nguyen, Nguyen Tat Thanh, Nguyen Van Vinh Chau, Guy Thwaites, John Perfect, Ruwanthi Kolamunnage-Dona, William Hope

**Affiliations:** a Division of Infectious Diseases and International Health, Duke University School of Medicine, Durham, North Carolina, USA; b Oxford University Clinical Research Unit, Ho Chi Minh City, Vietnam; c Hospital for Tropical Diseases, Ho Chi Minh City, Vietnam; d University of Medicine and Pharmacy, Ho Chi Minh City, Vietnam; e University of Houston College of Pharmacy, Houston, Texas, USA; f Nuffield Department of Medicine, University of Oxford, Oxford, United Kingdom; g Department of Biostatistics, Institute of Translational Medicine, University of Liverpool, Liverpool, United Kingdom; h Antimicrobial Pharmacodynamics and Therapeutics, Department of Molecular and Clinical Pharmacology, Institute of Translational Medicine, University of Liverpool, Liverpool, United Kingdom

**Keywords:** PK-PD, *Talaromyces*, amphotericin, antifungal, *Penicillium*, pharmacodynamics, population pharmacokinetics, talaromycosis

## Abstract

Amphotericin B deoxycholate (DAmB) is a first-line agent for the initial treatment of talaromycosis. However, little is known about the population pharmacokinetics and pharmacodynamics of DAmB for talaromycosis. Pharmacokinetic data were obtained from 78 patients; among them, 55 patients had serial fungal CFU counts in blood also available for analysis. A population pharmacokinetic-pharmacodynamic model was fitted to the data. The relationships between the area under the concentration-time curve (AUC)/MIC and the time to blood culture sterilization and the time to death were investigated. There was only modest pharmacokinetic variability in the average AUC, with a mean ± standard deviation of 11.51 ± 3.39 mg·h/liter. The maximal rate of drug-induced kill was 0.133 log_10_ CFU/ml/h, and the plasma concentration of the DAmB that induced the half-maximal rate of kill was 0.02 mg/liter. Fifty percent of patients sterilized their bloodstreams by 83.16 h (range, 13 to 264 h). A higher initial fungal burden was associated with a longer time to sterilization (hazard ratio [HR], 0.51; 95% confidence interval [CI], 0.36 to 0.70; *P* < 0.001). There was a weak relationship between AUC/MIC and the time to sterilization, although this did not reach statistical significance (HR, 1.03; 95% CI, 1.00 to 1.06, *P* = 0.091). Furthermore, there was no relationship between the AUC/MIC and time to death (HR, 0.97; 95% CI, 0.88 to 1.08; *P* = 0.607) or early fungicidal activity {slope = log[(0.500 − 0.003·(AUC/MIC)]; *P* = 0.319} adjusted for the initial fungal burden. The population pharmacokinetics of DAmB are surprisingly consistent. The time to sterilization of the bloodstream may be a useful pharmacodynamic endpoint for future studies. (This study has been registered at the ISRCTN registry under no. ISRCTN59144167.)

## Abstract (precis)

Amphotericin B deoxycholate (DAmB) is a first-line agent for the initial treatment of talaromycosis. However, little is known about the population pharmacokinetics and pharmacodynamics of DAmB for talaromycosis.

## INTRODUCTION

*Talaromyces* (formerly *Penicillium*) *marneffei* is a thermally dimorphic fungus that causes the systemic fungal infection talaromycosis (formerly penicilliosis). The organism is geographically restricted to South Asia, with cases being described in Vietnam, Thailand, India, and China. The bamboo rat and soil associated with bamboo rat burrows have been identified as animal and environmental reservoirs for *T. marneffei*, respectively (1). Human infection is most commonly presumed to be via inhalation of conidia in the environment (2). The majority of patients with talaromycosis have advanced HIV infection, but talaromycosis is emerging in other immunosuppressed populations (3). Disseminated disease is the most common form, with involvement of the skin, lungs, liver, spleen, lymph nodes, bone marrow, and bloodstream being characteristic (4). The rate of mortality is high and ranges from 11.3% to 29.4% at 6 months, despite the use of modern antifungal chemotherapy and supportive care (3, 5).

Amphotericin B deoxycholate (DAmB) is a highly potent and effective antifungal agent and is on the World Health Organisation’s list of essential medicines (http://www.who.int/medicines/publications/essentialmedicines/en/). This compound is widely used for induction therapy for cryptococcal meningoencephalitis (6) and has recently been demonstrated to be superior to itraconazole for the initial treatment of talaromycosis (5). There have been extensive studies that have examined the optimal dosage for invasive fungal infections caused by both yeasts and molds. Maximal antifungal activity is generally achieved with a dosage of 0.7 to 1 mg/kg of body weight/day (5), with no consistent evidence that dosages of >1 mg/kg/day result in better clinical outcomes for invasive fungal diseases. The toxicity of DAmB is well documented and in some circumstances is linked to suboptimal clinical outcomes (7, 8). Hence, there is a balance to strike between maximizing antifungal efficacy and minimizing drug-related toxicity.

Herein, we describe the population pharmacokinetics (PK) and pharmacodynamics (PD) of DAmB for patients with talaromycosis. These analyses were performed as a substudy of a recently published clinical trial that compared DAmB to itraconazole as induction therapy for talaromycosis (5). The data set was unique in that there were rich pharmacokinetic and pharmacodynamic data, as well as detailed information on clinical outcomes. An assessment of population pharmacodynamics was made possible by quantitation of the fungal counts within the bloodstream and an assessment of early fungicidal activity in a manner similar to that used for cryptococcal meningitis. The ability to explicitly link pharmacokinetics with real-time pharmacodynamic endpoints opens a multitude of opportunities for the optimization of antifungal regimens, the individualization of antifungal therapy, and the accelerated development of new antifungal agents for talaromycosis.

## RESULTS

### Study population.

The details about the 78 patients in this substudy are shown in Table 1. The mean age of the patients was 32 years (interquartile range [IQR], 27 to 37 years). All patients had advanced HIV infection with a median CD4 count of 7 cells per microliter (IQR, 4 to 14 cells per microliter). Sixty-eight percent of the patients were male. Patients were recruited between October 2012 and December 2015 from the Hospital for Tropical Diseases in Ho Chi Minh City, Vietnam. All patients had disseminated disease, with *Talaromyces marneffei* being isolated from blood, skin lesions, lymph nodes, and/or serous fluid.

**TABLE 1:** Clinical characteristics of PK patients and PK-PD patients^*a*^

Characteristic	Values for:	
	PK patients (*n *= 78)	PK and PD patients (*n *= 55)
No. of male patients/total no. of patients (%)	53/78 (67.9)	37/55 (67.2)
Median (IQR) age (yr)	32 (27–37)	31 (28–37)
Median (IQR) wt (kg)	45 (39–45)	44 (38–50)
Median (IQR) CD4^+^ T cell count (cells/mm^3^)	7 (4–14)	9 (4–15)
Median (IQR) creatinine concn (μmol/liter)	62 (52–80)	63 (54–81)
No. of patients with a blood culture positive for *T. marneffei*/total no. of patients (%)	57/78 (73.1)	55/55 (100)
Median (IQR) baseline fungal count (log_10_ CFU/ml) in blood culture-positive patients	2.53 (1.40–3.40)	2.53 (1.40–3.40)

a PK, pharmacokinetics; PD, pharmacodynamics.

### MICs.

MICs were determined using the M27-A3 reference methodology of the Clinical and Laboratory Standards Institute (CLSI) (9). The cumulative percentage of patient isolates for which MICs were 0.25, 0.5, and 1 mg/liter were 74, 79.6, and 100, respectively. Of the 55 patients in the pharmacokinetic-pharmacodynamic study, the MIC was not determined for the strain from 1 patient because it could not be resurrected from the frozen stock. Hence, the total number of strains was 54. There were 57 patients who had at least one isolate available for susceptibility testing, and the modal MIC value for these strains was 0.5 mg/liter (range, 0.25 to 1 mg/liter).

### Population pharmacokinetic modeling.

**FIG 1:**
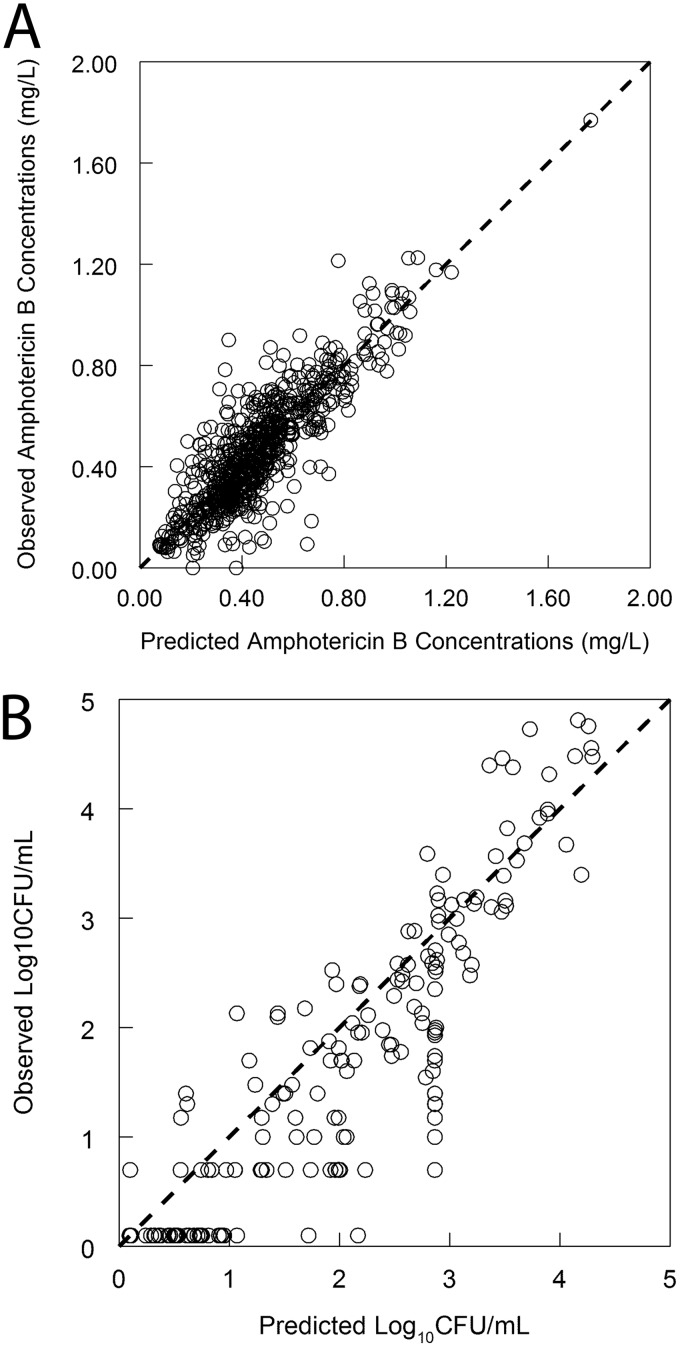
Observed-predicted values for the pharmacokinetic and pharmacodynamic models. The Bayesian posterior (i.e., individual predicted) values are shown in both panels. (A) Observed-predicted values for plasma concentrations of amphotericin B deoxycholate; (B) observed-predicted values for the fungal density in blood.

The base pharmacokinetic model fitted the data reasonably well, although there was a degree of bias both before and after the Bayesian step (data not shown). The parameter values for the base model are summarized in Table 2. There was a statistically significant relationship between weight and clearance, which provided the impetus to develop an expanded model that included the impact of weight on clearance. The expanded model resulted in less bias, better precision, and a statistically more likely result (as assessed using twice the difference in log likelihood values assessed against a chi-square distribution, *P* = 0.001). Furthermore, the coefficient of determination for the linear regression of observed-predicted values was improved, as were the estimates for the intercept (closer to zero) and slope (closer to 1) for the linear regression of the observed-predicted values. The parameter values for the final model are summarized in Table 3, and the observed-predicted values for the plasma concentrations of DAmB are shown in Fig. 1A.

**TABLE 2:** Parameter values for the base pharmacokinetic model

Parameter (units)^*a*^	Mean	Median	Standard deviation
SCL (liters/h)	2.485	2.575	1.055
*V_c_* (liters)	28.258	29.568	16.009
Kcp (h^−1^)	7.147	0.530	11.544
Kpc (h^−1^)	2.846	0.131	6.555
IC1 (mg)	3.071	2.974	1.930
IC2 (mg)	19.638	10.967	17.705

a SCL, clearance from the central compartment; *V_c_*, volume of the central compartment; Kcp and Kpc, first-order intercompartmental rate constants; IC1 and IC2, initial amounts of amphotericin B in the central and peripheral compartments, respectively.

**TABLE 3:** Parameter values for the final pharmacokinetic model for amphotericin B deoxycholate

Parameter^*a*^	Mean	Median	SD
Intercept (liters/h)	0.320	0.069	0.430
Slope	0.048	0.049	0.018
*V_c_* (liters)	23.907	22.650	13.836
Kcp (h^−1^)	8.996	1.001	12.812
Kpc (h^−1^)	4.237	0.184	8.003
IC1 (mg)	2.665	2.213	2.044
IC2 (mg)	16.059	8.061	19.355

a Clearance has been parameterized as intercept (in liters per hour) + slope (in liters per hour per kilogram)·weight (in kilograms). *V_c_* (in liters), volume of the central compartment; Kcp and Kpc, first-order intercompartmental rate constants; IC1 and IC2, the amounts of amphotericin B at the time of initiation of amphotericin B in the central and peripheral compartments, respectively.

**FIG 2:**
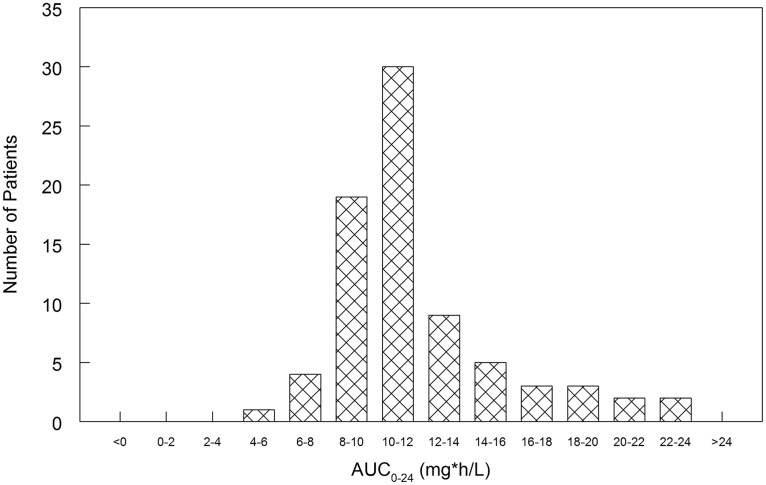
Histogram of the distribution of the average AUC_0–24_ for the 78 patients in the pharmacokinetic study (calculated as the entire AUC over the treatment course divided by the number of days of treatment). The mean ± standard deviation AUC was 11.84 ± 3.54 mg·h/liter, and the median AUC was 11.16 mg·h/liter.

The distribution of the average areas under the concentration-time curves (AUCs) for the 78 patients is shown in Fig. 2. The mean ± standard deviation for the average AUC from 0 to 24 h (AUC_0–24_) for patients receiving DAmB at 0.7 mg/kg/day was 11.84 ± 3.54 mg·h/liter, and the median was 11.16 mg·h/liter.

### Population pharmacodynamic modeling.

**FIG 3:**
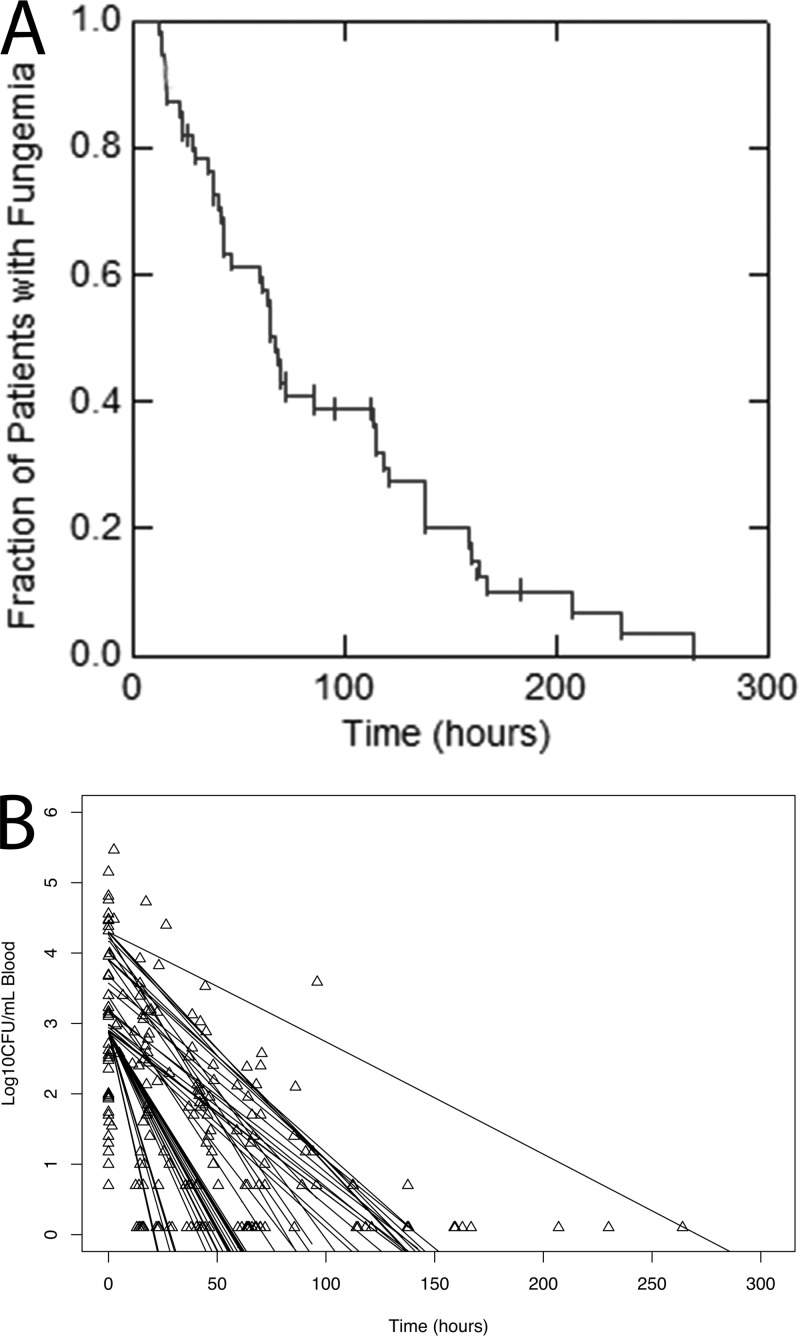
(A) Kaplan-Meier plot of the time to sterilization. (B) Time course of the reduction of the fungal burden for each of the 55 patients in the pharmacokinetic-pharmacodynamic study. The open triangles represent data points from individual patients, and the solid lines are the model estimates for each of the 55 individual patients.

The pharmacodynamics from 55 patients receiving DAmB with serial quantitative counts in the bloodstream were investigated using several pharmacodynamic structural models. A two-step process was used, where the pharmacokinetics (described above) were solved and the Bayesian estimates for each patient’s pharmacokinetic values were fixed. A pharmacodynamic model that described monophasic fungal killing in the bloodstream was used. For those patients with relatively rich pharmacodynamic data, it was clear that killing was monophasic until extinction (i.e., sterilization of the bloodstream) occurred or the counts dropped beneath a limit of detection. The parameter estimates from the population model are summarized in Table 4. In the majority of cases (48 out of 55 patients), there was rapid sterilization of the bloodstream, but in 7 patients there was persistent fungemia. The mean time to sterilization of the bloodstream was 83.16 h (range, 13 to 264 h) (Fig. 3). The model-predicted fungicidal activity of DAmB for the 55 patients superimposed on each patient’s data is shown in Fig. 3.

**TABLE 4:** Parameter values for the final pharmacodynamic model for amphotericin B deoxycholate against *Talaromyces marneffei*

Parameter^*a*^	Mean	Median	SD
Kkill_max_ (no. of log_10_ CFU/ml/h)	0.133	0.118	0.096
H	13.815	15.447	5.964
C50k (mg/liter)	0.016	0.010	0.016
IC (no. of CFU/ml)	4,318.564	733.770	7,560.737

a The model parameters are as follows: Kkill_max_ is the maximum rate of drug-induced killing of *Talaromyces marneffei*; H is the slope function (no units); C50k is the plasma concentration of amphotericin that induces the half-maximal rate of killing and IC is the estimated fungal density at the time of treatment initiation.

### Drug exposure versus probability of toxicity.

A total of 78 patients had pharmacokinetic data that were used to estimate the average AUC using a population pharmacokinetic approach. There was no relationship between the average AUC, the time to death, and other drug-related toxicities that are characteristic of DAmB over the 14 days of DAmB treatment. These included death (odds ratio [OR], 0.928; 95% confidence interval [CI], 0.74 to 1.16; *P* = 0.52), renal impairment (OR, 1.113; 95% CI, 0.894 to 1.385; *P* = 0.337), anemia (OR, 1.097; 95% CI, 0.959 to 1.254; *P* = 0.18), hypomagnesemia (OR, 1.126; 95% CI, 0.974 to 1.302; *P* = 0.110), and hypokalemia (OR, 0.878; 95% CI, 0.748 to 1.03; *P* = 0.110).

### Relationship between AUC/MIC and time to sterilization of the bloodstream.

**FIG 4:**
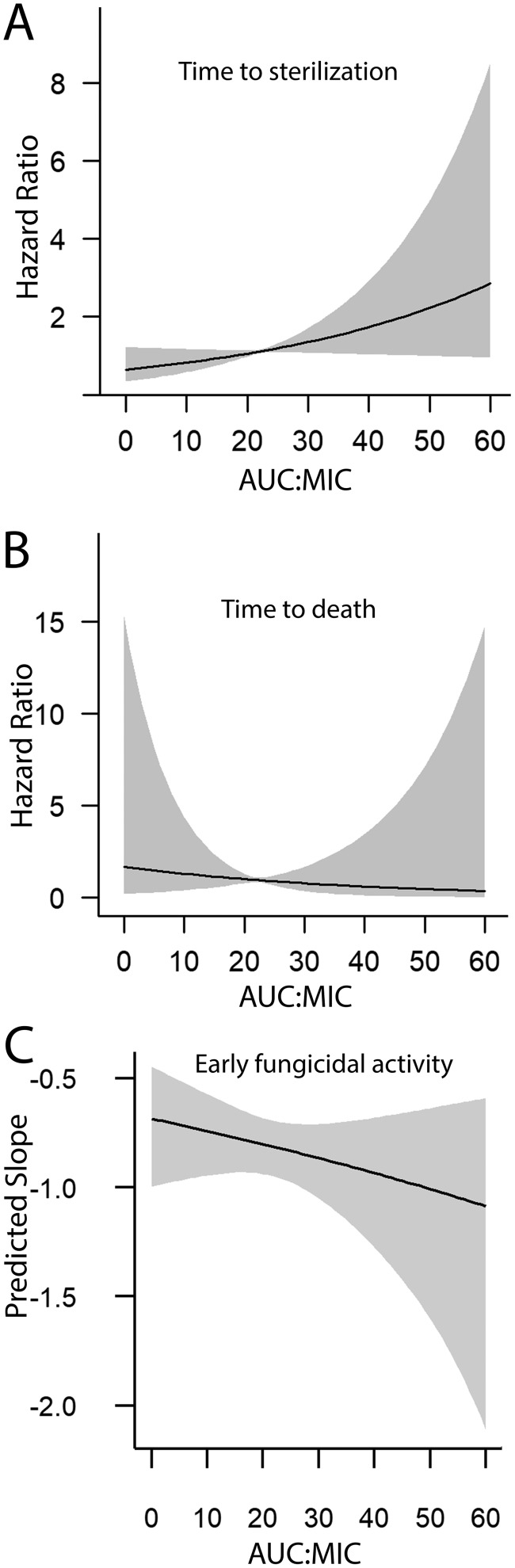
Predictions from the statistical models fitted to the pharmacodynamic data. (A) Cox model of AUC/MIC versus time to sterilization adjusted for the initial fungal burden. The change in the hazard ratio with AUC/MIC was estimated for the median initial fungal burden of 2.5 log_10_ CFU/ml. The hazard ratio adjusted for the initial fungal burden was 1.03 (95% CI, 1.00 to 1.06; *P* = 0.091). (B) Cox model of AUC/MIC versus time to death for the median initial fungal burden of 2.5 log_10_ CFU/ml. The hazard ratio adjusted for the initial fungal burden was 0.97 (95% CI, 0.88 to 1.08; *P* = 0.607). (C) Relationship between AUC/MIC and early fungicidal activity for the median initial fungal burden of 2.5 log_10_ CFU/ml such that the slope was equal to log[(0.500 − 0.003·(AUC/MIC)]. A one-unit increase in AUC/MIC decreases the exp(slope) by 0.003 units (0.3%) (95% CI, −0.008 to 0.003).

A Kaplan-Meir plot of the time to sterilization of the bloodstream is shown in Fig. 3. A total of 48 of 55 patients achieved sterilization of the bloodstream. A small number of patients had persistent fungemia, despite seemingly adequate drug exposure. The only variable that had an influence on the time to sterilization was the initial fungal burden (*P* < 0.001). Following adjustment for the initial fungal burden, there was a weak relationship between AUC/MIC and the time to sterilization, although this did not reach statistical significance (hazard ratio [HR], 1.03; 95% CI, 1.00 to 1.06; *P* = 0.09). There was no relationship between drug exposure (AUC/MIC) and the rate of decline of quantitative counts in the bloodstream (early fungicidal activity) {slope = log[(0.501 − 0.003·(AUC/MIC)]; *P* = 0.319} or the time to death (HR, 0.97; 95% CI, 0.88 to 1.08; *P* = 0.61). These relationships are summarized in Fig. 4.

## DISCUSSION

Our recent clinical trial reported that 73% of patients with disseminated talaromycosis were fungemic at presentation and that the involvement of multiple body sites was common (5). The fungemia provides an opportunity to describe the pharmacodynamics of DAmB and a potential way to assess new antifungal agents for this important fungal disease.

The estimates of the population PK parameters for DAmB in a previously conducted study are similar to those described in this study (10). Both studies used total rather than free concentrations of drug because of the complex binding properties of AmB. The coefficient of variation (CV) of the AUC for DAmB was recently estimated to be 44% for Vietnamese and Ugandan patients with cryptococcal meningitis (10). Similarly, in the current study, the average AUC varied by only 4- to 5-fold in patients receiving 0.7 mg/kg/day, which is remarkable for a group of patients who were otherwise heterogeneous in terms of fungal burden, illness severity, and extent of disease. The lack of variability in weight may partly contribute to the invariant drug exposure. Nevertheless, the PK of DAmB appear to be significantly less variable than those of other antifungal agents and classes, where significant variability in drug exposure is common. For example, the serum concentrations of voriconazole vary by 100-fold in healthy volunteers, and the variability is even greater in severely ill patients (11).

There was no statistically significant relationship between the AUC/MIC and the time to sterilization of the bloodstream (HR, 1.03; 95% CI, 1.00 to 1.06; *P* = 0.09). Perhaps a relationship would have been apparent with a larger number of patients. The majority of patients cleared their fungemia relatively quickly, with a median clearance time of 83.16 h (Fig. 3). Seven of 55 patients with positive blood cultures at the baseline had persistently positive blood cultures after 200 h of treatment, but this was not related to suboptimal drug exposure or reduced susceptibility to DAmB. The absence of any statistically significant link between drug exposure and various pharmacodynamic endpoints (i.e., various measures of drug-related toxicity, time to sterilization of blood cultures, early fungicidal activity, and time to death) is probably due to the induction of a nearly maximal antifungal effect in the majority of patients, the relatively tight variance structure around drug exposure (AUC), and a very narrow range of MICs of DAmB, which only ranged from 0.25 to 1 mg/liter (i.e., 2 doubling dilutions). In addition, there are inherent limitations of all the endpoints used in this study that may compromise the ability to resolve relevant PK-PD relationships. For example, mortality may be influenced by not only drug exposure but also comorbidities, multiorgan involvement, and the infectious burden.

The rate of decline of the fungal burden has been used extensively in phase II studies for cryptococcal meningitis (see, for example, reference 12). This has enabled the effect of a range of antifungal agents, regimens, and combinations to be assessed efficiently to facilitate the selection of candidate regimens before commencing late-staged clinical trials powered using mortality as a primary endpoint. Such trials typically require large sample sizes and extensive resources to conduct. The rate of decline of fungal density in the bloodstream was a secondary endpoint in our original clinical trial (5). The early fungicidal activity of DAmB was 4-fold faster than that observed for patients receiving itraconazole. In the current study, the time to sterilization of the bloodstream and mortality were also considered pharmacodynamic endpoints. Each of these three pharmacodynamic measures potentially provides complementary information, and none provides complete insight into the antifungal activity of DAmB in isolation.

The pharmacodynamics were challenging to describe and model. The usual approach to modeling such data is to consider both fungal growth and drug-induced fungal killing. In the current study, data on unrestrained fungal growth could not be collected and estimated because it is unethical to withhold treatment. Hence, only fungal killing was modeled, with the initial fungal burden being estimated as part of the fitting process. The decline in fungal burden was monoexponential, and the use of this function described the observed data well. There were some difficulties in modeling fungal counts that were zero because of uncertainty about whether this represented true sterilization or a fungal burden beneath a limit of detection. The spline fitted to the pooled pharmacodynamic data from all patients in the original publication gives the impression of a biphasic response with a rapid and slower phase of fungal killing (5). Plotting the decline of the fungal burden patient by patient suggests that the majority of patients (87%) have a rapid monoexponential decline and that the biphasic appearance is produced by a relatively small subset of patients that are slow to clear the fungi from their bloodstream. There was no hint of the emergence of drug resistance or the presence of a persistent (tolerant) subpopulation, although the latter was not formally examined.

This study is the first detailed pharmacokinetic-pharmacodynamic study of the antifungal treatment of talaromycosis and one of few that has described the population pharmacokinetics of DAmB. The insight that an early biomarker, such as the decline in the fungal burden in the bloodstream, may be used to assess the response to antifungal therapy is reminiscent of the similar use of galactomannan and the fungal burden for invasive aspergillosis and cryptococcal meningitis, respectively (13, 14). This biomarker could be exploited for clinical trials of new therapeutic approaches for talaromycosis, especially for phase II studies. Future studies could also examine the performance of other biomarkers, such as galactomannan and PCR, that may be useful for following the response to antifungal therapy. These biomarkers may circumvent some of the inherent imprecision in using the time to sterilization as an endpoint that results from intermittent sampling and the use of relatively small sample volumes to recover viable fungal propagules.

While DAmB has been shown to be a better agent than itraconazole for induction therapy of talaromycosis, a dose of 0.7 mg/kg/day also incurs significant toxicity. In the original clinical study, there were at least 25% of patients with grade III or greater adverse events that included infusional toxicity, azotemia, and electrolyte disturbances (5). Hence, innovative induction regimens with existing antifungal agents (e.g., combination antifungal therapy, abbreviated regimens) or the potential use of newer antifungal agents (e.g., F901318 [F2G, Eccles, UK], APX001 [Amplyx, San Diego, CA, USA]) should be studied. The use of serial quantitative counts provides a way of derisking such studies and rapidly identifying regimens that are associated with maximal antifungal activity for talaromycosis.

## MATERIALS AND METHODS

### Clinical study.

This pharmacokinetic-pharmacodynamic study was a substudy of a multicenter prospective randomized clinical trial that compared mortality and the clinical response to amphotericin B (0.7 mg/kg/day) and itraconazole at 200 mg every 12 h for induction therapy of HIV-associated talaromycosis (Itraconazole versus Amphotericin B for Talaromycosis [IVAP] trial). The trial protocol and results are published elsewhere (5). Briefly, patients >18 years of age were eligible for the study if they had evidence of HIV infection and culture-confirmed talaromycosis. Patients were excluded if they had central nervous system infection, had a positive urine pregnancy test, had liver transaminase levels of >400 U/liter, had absolute neutrophil counts of <500 cells/mm^3^, had a creatinine clearance of <30 ml/min, were taking a medication that may interact with itraconazole (e.g., rifampin), or had received antifungal therapy for >48 h. All patients who were enrolled into the IVAP trial at the Hospital for Tropical Diseases in Ho Chi Minh City were invited to participate in this pharmacokinetic-pharmacodynamic substudy. The substudy was approved by the ethics committees at the Hospital for Tropical Diseases, by the Oxford University Tropical Research Ethics Committee, and by the Vietnam Ministry of Health. The clinical trial registration number was ISRCTN59144167.

### *In vitro* suceptibility testing.

The MICs of amphotericin B (AmB) against *T. marneffei* were determined using the CLSI standardized M27-A3 methodology for yeasts (9). Briefly, a *T. marneffei* suspension was made in RPMI 1640 medium (Sigma, USA) to achieve the desired final inoculum size of 0.5 × 10^3^ to 2.5 × 10^3^ cells/ml. Candida krusei ATCC 6528 and Candida parasilopsis ATCC 22019 were used as the reference strains. The stock solution of DAmB (Sigma, USA) was prepared from a standard powder by dissolving it in 100% dimethyl sulfoxide (DMSO; Sigma, USA) and was serially 2-fold diluted in RPMI 1640 medium. Final drug concentrations ranged from 0.03 to 16 μg/ml. Drug solutions were inoculated onto 96-well microplates (Corning, USA) at 100 μl, followed by addition of 100 μl of the *T. marneffei* suspension for a final volume in each well of 200 μl. The microplates were then incubated at 37°C for 72 h. The MIC for DAmB was defined as the lowest concentration with no visible growth. All assays were performed in triplicate.

### Pharmacokinetic and pharmacodynamic studies and sampling.

Of the 86 patients that were randomized to receive DAmB at the Hospital for Tropical Diseases in the IVAP trial, 78 patients agreed to participate in the pharmacokinetic-pharmacodynamic substudy.

For pharmacokinetic sampling, a total of 2 ml of blood was collected and immediately placed on ice before being centrifuged at 2,000 rpm for 15 min. Heparized plasma was obtained and then frozen at −80°C until analysis. Samples were processed within 30 min of blood collection. Drug was extracted on site in Ho Chi Minh City (see below), before being shipped to the University of Liverpool for analysis. Samples mixed with acetonitrile were plated onto Sabouraud agar to confirm sterility in three independently conducted experiments that were conducted at the Oxford University Clinical Research Unit (OCCRU) in Ho Chi Minh City (data not shown).

For pharmacodynamic sampling, blood was collected for quantitative cultures daily for the first 4 days and then every other day thereafter over the first 14 days until the cultures turned negative. A total of 100 μl of blood was serially diluted 10-fold and then plated onto Sabouraud dextrose agar. The plates were incubated at 37°C for CFU quantification.

### Bioanalytical methodology.

Amphotericin B concentrations in plasma were measured using high-performance liquid chromatography (HPLC) with a Shimadzu Prominence HPLC system (Shimadzu, Milton Keynes, UK). Amphotericin B was extracted by protein precipitation. A total of 300 μl of methanol that contained piroxicam at 2 mg/liter (Sigma-Aldrich, Dorset, UK) as the internal standard was added to 100 μl of matrix. Samples were vortexed for 5 s and then centrifuged at 13,000 × *g* for 3 min.

One hundred fifty microliters of supernatant was removed and placed in a 96-well plate, to which 50 μl of water was added. A 50-μl aliquot was injected onto a Kinetex 5-μm-particle-size XB-C_18_ liquid chromatography column (Phenomenex, Macclesfield, UK). Chromatographic separation was achieved using a gradient with the starting conditions of 75% mobile phase A and 25% mobile phase B (0.1% formic acid in water as mobile phase A and 0.1% formic acid in acetonitrile as mobile phase B). Mobile phase B was increased to 80% over 5 min and then reduced to the starting conditions for 2 min of equilibration. Amphotericin B and the internal standard were detected using UV detection at wavelengths of 406 nm and 385 nm, respectively; they eluted after 4.1 and 4.6 min, respectively.

The standard curve for amphotericin B encompassed a concentration range of 0.05 to 8.0 mg/liter and was constructed using a blank matrix. The limit of quantitation was 0.05 mg/liter. The coefficient of variation was <9.3% over the concentration range of 0.05 to 8 mg/liter. The intra- and interday variation was <7.9%.

### Population pharmacokinetic modeling.

A population methodology was used for all fitting, and the population pharmacokinetic program Pmetrics was used for this purpose (15). As previously described (16), a two-step process was used, whereby the population pharmacokinetics of DAmB were estimated before solving the population pharmacodynamics. This approach was used to provide stability for the estimation of the pharmacodynamics.

For the population pharmacokinetic model, a standard 2-compartment base model that consisted of central and peripheral compartments was used. There was time-limited zero-order input (infusion) of DAmB into the central compartment (i.e., bloodstream) with first-order clearance of drug from the central compartment. The central and peripheral compartments were connected by two first-order intercompartmental rate constants that described the transfer of mass back and forth between the two compartments (Kcp and Kpc, respectively). Because some patients (15/78; 19.2%) had DAmB started before being enrolled in the study, their initial pharmacokinetic sample showed quantifiable concentrations of drug. This was handled by estimating the amount of drug (in milligrams) in the central and peripheral compartments at the initiation of treatment. To achieve this, a switch was written, which defaulted to zero if sampling commenced prior to drug administration. This in turn defaulted the amount of drug in the compartments at time zero to zero.

The base model was as follows: XP(1) = *R*(1) − (SCL/*V_c_*) × *X*(1) + Kpc × *X*(2) and XP(2) = Kcp × *X*(1) − Kcp × *X*(2), where XP(1) and XP(2) are the rate of change of the DAmB mass (in milligrams) in the central and peripheral compartments, respectively. Similarly, *X*(1) and *X*(2) represent the mass (in milligrams) of DAmB in the central and peripheral compartments, respectively. *R*(1) is the infusion of DAmB; SCL is the first-order clearance of DAmB from the central compartment, *V_c_* is the volume of the central compartment; Kpc and Kpc are the first-order intercompartmental rate constants. The output equation to estimate the concentration-time profile of DAmB was given by *Y*(1) = *X*(1)/*V_c_*.

The Bayesian estimates of volume and clearance were obtained and plotted against weight, which was the only clinically relevant covariate that was available for analysis. A linear regression of clearance versus weight was performed. Since this was statistically significant, clearance was reparametrized as intercept + weight·slope, with the units for intercept and slope being liters per hour and liters per hour per kilogram, respectively.

The final model was as follows: XP(1) = *R*(1) − [(int + slope · weight)/*V_c_*] × *X*(1) + Kpc × *X*(2) and XP(2) = Kcp × *X*(1) − Kcp × *X*(2), where int is the intercept.

The final model was refitted to the data.

The fit of each model to the data was assessed by estimating bias and precision, a visual inspection of the linear regression of the observed-predicted plots, both before and after the Bayesian step. Models were distinguished by determining twice the difference in log-likelihood values following the convergence of each model and comparing that value against a chi-square distribution with the suitable number of degrees of freedom (i.e., the difference in parameter number between the respective models).

The AUC was calculated as the average AUC (i.e., the total AUC for the treatment course divided by the number of 24-h intervals for the regimen). Such an approach was necessary because of the real-world nature of the data, where the precise administration times for DAmB varied from patient to patient and prevented estimates of drug exposure in convenient time intervals. The AUC was estimated using the trapezoidal rule in Pmetrics from each patient’s posterior mean parameter estimates.

### Pharmacokinetic and pharmacodynamic modeling.

The Bayesian estimates for each patient’s pharmacokinetics were obtained from the population model described above. An estimate of the weighting functions for the pharmacodynamics was required. To achieve this, the maximum likelihood estimator in ADAPT 5 was used. The following variance model was used: variance = [intercept + slope × *Y*(1)]^2^.

The Bayesian posterior estimates for the pharmacokinetic parameters that had been previously estimated from the pharmacokinetic model were fixed for each patient. The individual’s pharmacodynamic data were then entered, and the following structural model was fitted to the pharmacodynamic data one patient at a time.

$$\frac{dN}{dt}=-{\text{Kkill}}_{\max}\times \frac{\frac{X{\left(1\right)}^{H}}{V_{c}}}{\frac{X{\left(1\right)}^{H}}{V_{c}}+{\text{C}50\text{k}}_{50}^{H}}\times N$$

where *dN*/*dt* represents the rate of change of the organism in the bloodstream (where *N* is the number of CFU and *t* is time). The pharmacodynamic parameters (Kkill_max_, C50k_50_, and *H*, which are the maximal rate of fungal kill, the concentration of DAmB that induces half maximal rate of killing, and the slope function, respectively) and initial condition (the fungal density in blood just prior to the commencement of DAmB) were estimated along with the parameters for the variance model (i.e., the intercept and slope), as described above. The weighting estimates for each patient were then resupplied to Pmetrics to enable the population pharmacodynamic problem to be solved. The fit of the final pharmacodynamic model to the data was assessed in the same way as described above for the pharmacokinetics. The final pharmacodynamic model was then used to estimate the Bayesian posterior estimates for the pharmacodynamics. The pharmacokinetic and pharmacodynamic models enabled the link between the dose of DAmB, the drug exposure (quantified in terms of the AUC/MIC), and the time course of antifungal activity in the bloodstream to be determined.

### Statistical modeling.

Potential relationships between patients in the pharmacokinetic study who had estimates of AUCs available and the probability of toxicity were explored using logistic regression. Outcome measures were death at 6 months, a grade 2 rise in the creatinine concentration, grade 2 anemia, grade 2 hypomagnesemia, and grade 2 hypokalemia.

Cox proportional hazard models were fitted to determine the effect of AUC/MIC on the time to sterilization and the time to death. Models were adjusted for initial fungal burden and took the form $\lambda \left(t\right)=\lambda_{0}\left(t\right)\exp\left(\frac{\beta_{1}\text{AUC}}{\text{MIC}}+\beta_{2}\text{initial}\;\text{fungal}\;\text{burden}\right)$, where λ(*t*) is the hazard and λ_0_(*t*) is the baseline hazard. β_1_ and β_2_ are the coefficients for regression and exp(β) provides an estimate of the hazard ratio (HR). The proportional hazards assumption was tested using weighted residuals for the time-dependent effect of AUC/MIC.

To assess the relationship between AUC/MIC and the rate of decline of the fungal burden (early fungicidal activity or slope), exp(slope) was fitted in a linear model of the form $\text{exp}(\text{slope})=\beta_{0}+\beta_{1}\frac{\text{AUC}}{\text{MIC}}+\beta_{2}\;\text{initial fungal burden}+\varepsilon$, where ε is the model error term, and β_0_ and β_1_ and β_2_ are the intercept and coefficients for the regression. The exponentially transformed slope provided better model diagnostics.

## References

[1] Huang X, He G, Lu S, Liang Y, Xi L 2015 Role of Rhizomys pruinosus as a natural animal host of Penicillium marneffei in Guangdong, China. Microb Biotechnol 8:659–664. doi:10.1111/1751-7915.12275. 25824250 PMC4476820

[2] Vanittanakom N, Cooper CR, Fisher MC, Sirisanthana T 2006 Penicillium marneffei infection and recent advances in the epidemiology and molecular biology aspects. Clin Microbiol Rev 19:95–110. doi:10.1128/CMR.19.1.95-110.2006. 16418525 PMC1360277

[3] Chan JFW, Lau SKP, Yuen KY, Woo PCY 2016 Talaromyces (Penicillium) marneffei infection in non-HIV-infected patients. Emerg Microbes Infect 5:e19. doi:10.1038/emi.2016.18. 26956447 PMC4820671

[4] Le T, Wolbers M, Chi NH, Quang VM, Chinh NT, Lan NPH, Lam PS, Kozal MJ, Shikuma CM, Day JN, Farrar J 2011 Epidemiology, seasonality, and predictors of outcome of AIDS-associated Penicillium marneffei infection in Ho Chi Minh City, Viet Nam. Clin Infect Dis 52:945–952. doi:10.1093/cid/cir028. 21427403 PMC3106230

[5] Le T, Kinh NV, Cuc NTK, Tung NLN, Lam NT, Thuy PTT, Cuong DD, Phuc PTH, Vinh VH, Hanh DTH, Tam VV, Thanh NT, Thuy TP, Hang NT, Long HB, Nhan HT, Wertheim HFL, Merson L, Shikuma C, Day JN, Chau NVV, Farrar J, Thwaites G, Wolbers M 2017 A trial of itraconazole or amphotericin B for HIV-associated talaromycosis. N Engl J Med 376:2329–2340. doi:10.1056/NEJMoa1613306. 28614691

[6] Molloy SF, Kanyama C, Heyderman RS, Loyse A, Kouanfack C, Chanda D, Mfinanga S, Temfack E, Lakhi S, Lesikari S, Chan AK, Stone N, Kalata N, Karunaharan N, Gaskell K, Peirse M, Ellis J, Chawinga C, Lontsi S, Ndong J-G, Bright P, Lupiya D, Chen T, Bradley J, Adams J, van der Horst C, van Oosterhout JJ, Sini V, Mapoure YN, Mwaba P, Bicanic T, Lalloo DG, Wang D, Hosseinipour MC, Lortholary O, Jaffar S, Harrison TS, ACTA Trial Study Team. 2018 Antifungal combinations for treatment of cryptococcal meningitis in Africa. N Engl J Med 378:1004–1017. doi:10.1056/NEJMoa1710922. 29539274

[7] Wingard JR, Kubilis P, Lee L, Yee G, White M, Walshe L, Bowden R, Anaissie E, Hiemenz J, Lister J 1999 Clinical significance of nephrotoxicity in patients treated with amphotericin B for suspected or proven aspergillosis. Clin Infect Dis 29:1402–1407. doi:10.1086/313498. 10585786

[8] Bicanic T, Bottomley C, Loyse A, Brouwer AE, Muzoora C, Taseera K, Jackson A, Phulusa J, Hosseinipour MC, Van Der Horst C, Limmathurotsakul D, White NJ, Wilson D, Wood R, Meintjes G, Harrison TS, Jarvis JN 2015 Toxicity of amphotericin B deoxycholate-based induction therapy in patients with HIV-associated cryptococcal meningitis. Antimicrob Agents Chemother 59:7224–7231. doi:10.1128/AAC.01698-15. 26349818 PMC4649151

[9] Clinical and Laboratory Standards Institute. 2009 Reference method for broth dilution antifungal susceptibility testing of filamentous fungi, approved standard, 2nd ed Clinical and Laboratory Standards Institute, Wayne, PA.

[10] Stott KE, Beardsley J, Whalley S, Kibengo FM, Mai NTH, Kolamunnage-Dona R, Hope W, Day J 2018 Amphotericin B deoxycholate in adults with cryptococcal meningitis; a population pharmacokinetic model and meta-analysis of outcomes. Antimicrob Agents Chemother 62:e02526-17. doi:10.1128/AAC.02526-17. 29735567 PMC6021666

[11] Hope WW 2012 Population pharmacokinetics of voriconazole in adults. Antimicrob Agents Chemother 56:526–531. doi:10.1128/AAC.00702-11. 22064545 PMC3256045

[12] Jarvis JN, Leeme TB, Molefi M, Chofle AA, Bidwell G, Tsholo K, Tlhako N, Mawoko N, Patel RKK, Tenforde MW, Muthoga C, Bisson GP, Kidola J, Changalucha J, Lawrence D, Jaffar S, Hope W, Molloy SF, Harrison TS 26 June 2018 Short course high-dose liposomal amphotericin B for HIV-associated cryptococcal meningitis: a phase-II randomized controlled trial. Clin Infect Dis. doi:10.1093/cid/ciy515. PMC6336908 29945252

[13] Negri CE, Johnson A, McEntee L, Box H, Whalley S, Schwartz JA, Ramos-Martín V, Livermore J, Kolamunnage-Dona R, Colombo AL, Hope WW 2018 Pharmacodynamics of the novel antifungal agent F901318 for acute sinopulmonary aspergillosis caused by Aspergillus flavus. J Infect Dis 217:1118–1127. doi:10.1093/infdis/jix479. 28968675 PMC5909626

[14] Kovanda L, Kolamunnage-Dona R, Neely M, Maertens J, Lee M, Hope W 2017 Pharmacodynamics of isavuconazole for invasive mold disease: role of galactomannan for real-time monitoring of therapeutic response. Clin Infect Dis 64:1557–1563. doi:10.1093/cid/cix198. 28472247 PMC5434340

[15] Neely MN, van Guilder MG, Yamada WM, Schumitzky A, Jelliffe RW 2012 Accurate detection of outliers and subpopulations with Pmetrics, a nonparametric and parametric pharmacometric modeling and simulation package for R. Ther Drug Monit 34:467–476. doi:10.1097/FTD.0b013e31825c4ba6. 22722776 PMC3394880

[16] Huurneman LJ, Neely M, Veringa A, Pérez FD, Ramos-Martin V, Tissing WJ, Alffenaar JWC, Hope W 2016 Pharmacodynamics of voriconazole in children: further steps along the path to true individualized therapy. Antimicrob Agents Chemother 60:2336–2342. doi:10.1128/AAC.03023-15. 26833158 PMC4808208

